# GJA1 Expression and Its Prognostic Value in Cervical Cancer

**DOI:** 10.1155/2020/8827920

**Published:** 2020-11-24

**Authors:** Silu Meng, Xinran Fan, Jianwei Zhang, Ran An, Shuang Li

**Affiliations:** ^1^Department of Obstetrics and Gynecology, Tongji Hospital, Tongji Medical College, Huazhong University of Science and Technology, Wuhan, Hubei 430030, China; ^2^Department of Dermatology, Tongji Hospital, Tongji Medical College, Huazhong University of Science and Technology, Wuhan, Hubei 430030, China

## Abstract

Gap Junction Protein Alpha 1 (GJA1) belongs to the gap junction family and has been widely studied in cancers. We evaluated the role of GJA1 in cervical cancer (CC) using public data from The Cancer Genome Atlas (TCGA) and the Gene Expression Omnibus (GEO) database. The difference of GJA1 expression level between CC and normal tissues was analyzed by the Gene Expression Profiling Interactive Analysis (GEPIA), six GEO datasets, and the Human Protein Atlas (HPA). The relationship between clinicopathological features and GJA1 expression was analyzed by the chi-squared test and the logistic regression. Kaplan–Meier survival analysis and Cox proportional hazard regression analysis were used to assessing the effect of GJA1 expression on survival. Gene set enrichment analysis (GSEA) was used to screen the signaling pathways regulated by GJA1. Immune Cell Abundance Identifier (ImmuCellAI) was chosen to analyze the immune cells affected by GJA1. The expression of GJA1 in CC was significantly lower than that in normal tissues based on the GEPIA, GEO datasets, and HPA. Both the chi-squared test and the logistic regression showed that high-GJA1 expression was significantly correlated with keratinization, hormone use, tumor size, and FIGO stage. The Kaplan–Meier curves suggested that high-GJA1 expression could indicate poor prognosis (*p* = 0.0058). Multivariate analysis showed that high-GJA1 expression was an independent predictor of poor overall survival (HR, 4.084; 95% CI, 1.354-12.320; *p* = 0.013). GSEA showed many cancer-related pathways, such as the p53 signaling pathway and the Wnt signaling pathway, were enriched in the high-GJA1-expression group. Immune cell abundance analysis revealed that the abundance of CD8 naive, DC, and neutrophil was significantly increased in the high-GJA1-expression group. In conclusion, GJA1 can be regarded as a potential prognostic marker of poor survival and therapeutic target in CC. Moreover, many cancer-related pathways may be the critical pathways regulated by GJA1. Furthermore, GJA1 can affect the abundance of immune cells.

## 1. Introduction

Cervical cancer (CC) is the fourth most common cancer in females, with 570,000 new cases and 311,000 deaths estimated for 2018 worldwide [1]. The vast majority of CC is in sub-Saharan Africa and South-Eastern Asia, and there were 98,900 new cases and 30,500 deaths for 2015 in China [1, 2]. Although the incidence and mortality rate of cervical declined due to vaccination, screening, and the control of precancerous lesions, it is even higher in some rural areas in China with the characteristics of young age (peak 45-49) and late stage [3–5]. In clinical, patients with early-stage CC (stages I to IIA) are mainly treated with surgery, whereas those with late-stage CC (stages IIB to IV) are treated with chemoradiotherapy [6]. However, the recurrence rate of CC is approximately 20%–25%, and the 5-year relative survival rate of CC remains poor in China (overall 45.4%), and the data from a retrospective population-based study in Hong Kong also showed that 5-year relative survival rates were 90.9%, 71.0%, 41.7%, and only 7.8% for the stages I, II, III, and IV, respectively [7–9]. Therefore, it is crucial to identify sensitive and specific biomarkers that could predict CC prognosis and serve as a target for CC treatment.

GJA1 maps to 6q22.31. GJA1 is also known as Connexin43 and belongs to the gap junction family [10]. Gap junction family plays an essential role in determining cellular phenotypes [10]. The role of gap junction family in cancers has been widely studied, among which GJA1 is one of the most studied genes. Firstly, GJA1 has been reported to play a suppressive role in tumorigenesis [11–13]. GJA1 could facilitate the transmission of cAMP, leading to increased p27 levels and reduced tumor growth by gap junctional intercellular communication [11]. Moreover, GJA1 could inhibit cell proliferation by inhibiting the Wnt signaling pathway [12, 13]. Secondly, although GJA1 has suppressive effects on cancer, many articles have reported the overexpression of GJA1 in metastatic tumors [14, 15]. Thirdly, GJA1 could also accelerate cancer progression by promoting cell migration via activating p38 [16, 17]. Also, high-GJA1 mRNA expression levels could indicate a more unsatisfactory outcome in cancer [18].

Based on the TCGA database and the GEO database, we firstly explored the difference of GJA1 expression level between CC and normal tissues. Also, we analyzed the relationship between GJA1 expression and the clinicopathological features of patients with CC and its prognostic value in CC. To gain further insight into the biological pathways and immune cell changes involved in CC pathogenesis related to GJA1, GSEA and immune cell abundance analysis were performed.

## 2. Materials and Methods

### 2.1. RNA-Sequencing Patient Data in the TCGA Database

The gene expression data (304 cases, workflow type: HTSeq-FPKM-UQ) and corresponding clinical information were downloaded from TCGA database (https://portal.gdc.cancer.gov) by TCGAbiolinks package [19]. The details of clinical information are shown in Table 1. Besides, it should be emphasized that survival information, including vital status and overall survival (OS) time, was directly taken from the articles of Liu et al. published in *Cell* in 2018 [20].

### 2.2. Relative GJA1 Expression Level between CC and Normal Tissues

GEPIA (http://gepia.cancer-pku.cn/) was used to access the mRNA expression levels of GJA1 in CC (based on TCGA database) and normal tissues (based on GTEx project) [21]. limma package [22] was used to explore whether the expression of GJA1 is significantly different between CC and normal tissues in six GEO datasets: GSE39001 [23], GSE52903 [24], GSE63514 [25], GSE6791 [26], GSE7803 [27], and GSE9705 [28]. Meeting the criteria of |LogFC | >1 and adjusted *p* value < 0.05, GJA1 could be considered differentially expressed between CC and normal tissues. Additionally, we explored the expression of GJA1 at the protein level by the HPA database [29].

### 2.3. Survival Analysis

We matched the GJA1 expression data with survival information derived from Liu et al. [20]. The patients in cohorts were divided into two groups according to the median expression levels of GJA1 (the low-GJA1-expression group and the high-GJA1-expression group). The survival and survminer packages were used for survival analysis and visualization, and then the Kaplan-Meier survival curve was obtained. Also, we conducted a stratified analysis and explored the prognostic value of GJA1 in squamous cell carcinoma (SCC) patients, early-stage patients (FIGO stage ≤ IIA2), and late-stage patients (FIGO stage ≥ IIB).

### 2.4. Univariate and Multivariate Cox Regression Analyses

The Cox proportional hazard regression model was used to conduct univariate and multivariate analyses. The hazard ratio (HR) value and 95% confidence interval (CI) were calculated. In univariate analysis, the independent predictive value of the clinicopathological parameters and GJA1 expression on survival was evaluated. Multivariate Cox analysis was conducted to compare the influence of GJA1 expression on survival along with other characteristics as categorical variables. Variables with *p* value ≤ 0.05 or close to 0.05, including age, BMI, tumor size, lymph node, FIGO stage, lymphovascular invasion, and distant metastasis, were entered in the multivariate Cox regression analysis as categorical variables. The cut-off value of GJA1 expression was set based on the median expression value. The data were analyzed by using survival and survminer packages and visualized by forestplot package in R.

### 2.5. Gene Set Enrichment Analysis (GSEA)

GSEA (version 4.0.3) was used to explore the signaling pathways related to GJA1 in cervical cancer [30]. Gene expression enrichment analysis was carried out between different phenotypes determined by the GJA1 expression level. The annotated gene set was selected (c2.cp.kegg.v7.1.symbols.gmt) as the reference gene set. Gene set permutations were performed 1,000 times for analysis. The normalized enrichment score (NES), nominal *p* value, and false discovery rate (FDR) *q*-value were used to sort the pathways enriched in each group. Pathways with NES > 1, nominal *p* value < 0.05, and FDR *q* − value < 0.25 were selected out.

### 2.6. Immune Cell Abundance Analysis

ImmuCellAI is a tool to estimate the abundance of 24 immune cells from gene expression datasets [31]. We used this tool to survey the infiltration difference of immune cells between the low- and the high-GJA1 expression groups.

### 2.7. Statistical Analysis

All statistical analyses were performed with IBM SPSS statistical software (version 26.0) and R software (version 3.6.3), and *p* < 0.05 was used to be as the significance level. The differences in GJA1 expression between the two groups were compared by Wilcoxon test. Chi-squared (*χ*^2^) test and logistic regression analysis were used to evaluate the relationship between GJA1 expression and clinicopathological parameters. Kaplan–Meier analysis and log-rank test were used to explore the relationship between survival rates and the GJA1 expression level. A Cox proportional hazard regression model was used for univariate and multivariate survival analysis.

## 3. Results

### 3.1. The Difference of GJA1 Expression between CC and normal tissues

**Figure 1:**
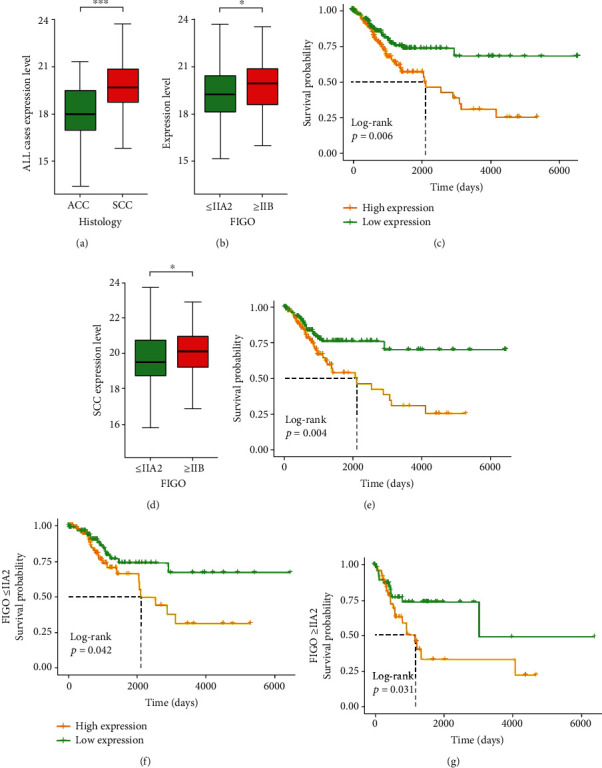
The expression of GJA1 and its association with clinicopathological parameters based on TCGA data: (a) histology type; (b) FIGO stage of all CC patients; (c) impact of GJA1 expression on OS in all CC patients; (d) FIGO stage of SCC patients; (e) impact of GJA1 expression on OS in all SCC patients; (f) impact of GJA1 expression on OS in patients with FIGO stage ≤ IIA2; (g) impact of GJA1 expression on OS in patients with FIGO stage ≥ IIB. TCGA: The Cancer Genome Atlas; FIGO: the International Federation of Gynecology and Obstetrics; CC: cervical cancer; OS: overall survival; SCC: squamous cell carcinoma; ACC: adenomas and adenocarcinomas; GJA1: gap junction protein alpha 1; ^∗^*p* < 0.05, ^∗∗^*p* < 0.01, and ^∗∗∗^*p* < 0.001.

GEPIA was used to explore the mRNA expression levels of GJA1 in CC and normal tissues, showing that GJA1 expression is significantly decreased in CC tissues (Figure S1A). Similar results were observed in five of the six GEO datasets.(Table S1). On the protein level, the expression of GJA1 was also higher in normal tissues than in CC tissues by using the HPA database (Figure S1B). In addition, the expression level of GJA1 was different in groups classified according to histology (*p* < 0.001, Figure 1(a)) and FIGO stage (*p* = 0.018, Figure 1(b)). In a stratified analysis of SCC patients, we found the same results that the GJA1 expression level was related to the FIGO stage (*p* = 0.038, Figure 1(d)).

### 3.2. High Expression of GJA1 in CC Is Related to Poor Overall Survival

We evaluated the relationship between GJA1 expression and prognosis of CC patients by Kaplan–Meier risk estimates. Compared to the low-GJA1 expression, the high-GJA1 expression was significantly associated with poor overall survival (*p* = 0.006, Figure 1(c)). The median OS of the high-GJA1-expression group was 627.5 days (range: 0-5271 days), while for the low-GJA1-expression group, the median OS was 734.5 days (range: 0-6408 days). The 5-year survival rate of patients in the low-GJA1-expression group (32.9%) was also higher than that in the high-GJA1-expression group (25.0%). In the stratified analysis of SCC patients, early-stage patients (FIGO ≤ IIA2), and late-stage patients (FIGO ≥ IIB), the high-GJA1 expression group all showed poor prognosis, and the *p* value was 0.004, 0.042, and 0.031, respectively **(**Figures 1(e)–1(g)**)**.

### 3.3. The relationship between GJA1 Expression and Clinicopathological Variables

To further explore the relationship between GJA1 expression and clinicopathological parameters, the clinical data of 304 CC samples with GJA1 expression data were analyzed from the TCGA database. Using chi-squared (*χ*^2^) test, the high expression level of GJA1 was significantly correlated with keratinization (*p* = 0.045), hormone use (*p* = 0.019), tumor size (0.042), and FIGO stage (*p* = 0.003) **(**Table 2**)**. Multiple logistic regression analysis showed that the increased expression of GJA1 in CC was significantly correlated with keratinization (OR, 1.987; 95% CI, 1.011-3.905; *p* = 0.046), hormone use (OR, 0.457; 95% CI, 0.236-0.885; *p* = 0.020), tumor size (OR, 2.234; 95% CI, 1.013-4.927; *p* = 0.046), and FIGO stage (OR, 2.062; 95% CI, 1.274-3.338; *p* = 0.003) **(**Table 3**)**.

### 3.4. The Effect of GJA1 Expression on Survival Based on Univariate and Multivariate Analyses

**Figure 2:**
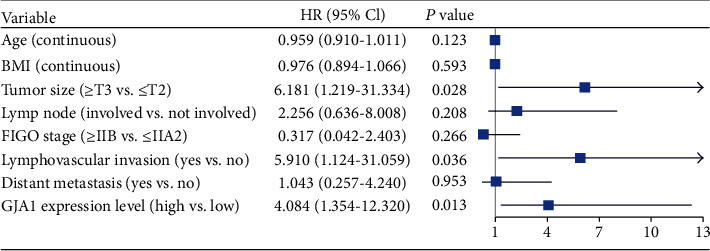
Forest plot for the multivariate Cox proportional hazard regression model. High-GJA1 expression was an independent predictor of poor survival rate (HR, 4.084; 95% CI, 1.354-12.320; *p* = 0.013). GJA1: Gap Junction Protein Alpha 1; HR: hazard ratio; CI: confidence interval; ^∗^*p* < 0.05, ^∗∗^*p* < 0.01, and ^∗∗∗^*p* < 0.001.

The univariate and multivariate Cox proportional hazard regression analyses were performed to investigate whether the high-GJA1 expression is an independent predictor of poor survival in CC patients. After excluding patients with incomplete data, we included 120 patients with CC for the multivariate Cox proportional hazard regression analysis. The univariate analysis showed that BMI (HR, 0.953; 95% CI, 0.911-0.996; *p* = 0.034), tumor size (HR, 3.643, 95% CI, 1.922-6.904, *p* < 0.001), lymph node (HR, 2.695; 95% CI, 1.358-5.349; *p* = 0.0046), FIGO stage (HR, 1.861; 95% CI, 1.166-2.970; *p* = 0.009), lymphovascular invasion (HR, 10.041; 95% CI, 2.361-42.700; *p* = 0.002), distant metastasis (HR, 3.141; 95% CI, 1.866-5.289; *p* < 0.001), and high-GJA1 expression (HR, 1.943; 95% CI, 1.202-3.140; *p* = 0.007) were important predictors of survival (Table 4). As the *p* value is close to 0.05, age was also included in multivariate analysis. The results showed that tumor size (HR, 6.181; 95% CI, 1.219-31.334; *p* = 0.028), lymphovascular invasion (HR, 5.910; 95% CI, 1.124-31.059; *p* = 0.036), and the high-GJA1 expression (HR, 4.084; 95% CI, 1.354-12.320; *p* = 0.013) were the important independent predictors of poor overall survival of CC (Figure 2 and Table 4).

### 3.5. Identification of GJA1-Related Signaling Pathways by GSEA

**Figure 3:**
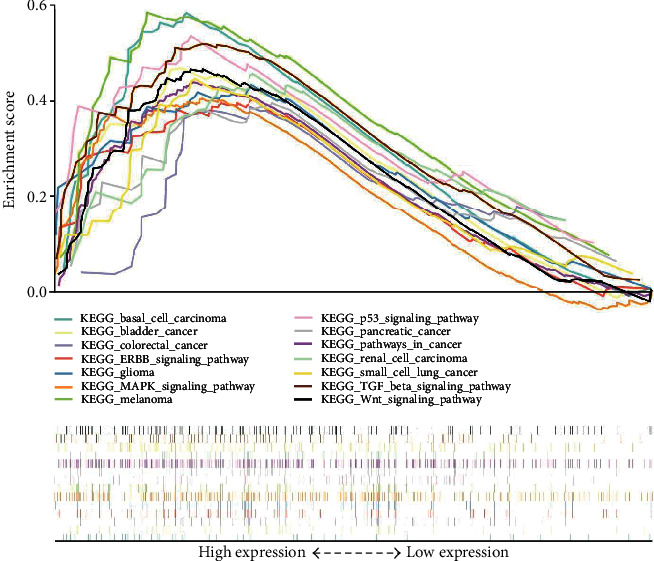
A merged enrichment plot from gene set enrichment analysis (GSEA) including enrichment score and gene sets. 14 cancer-related pathways are shown here.

To identify signaling pathways that are differentially activated in CC, we conducted the Gene Set Enrichment Analysis (GSEA) between low- and high-GJA1-expression datasets. We selected out 30 significantly enriched signaling pathways based on the standard that is NES > 1, nominal *p* value < 0.05, and FDR *q* − value < 0.25 (Table S2).  Figure 3 shows 14 cancer-related pathways enriched in high-GJA1-expression groups, including many well-known pathways, such as the P53 signaling pathway, the Wnt signaling pathway, and the MAPK signaling pathway.

### 3.6. Immune Cell Abundance Analysis

**Figure 4:**
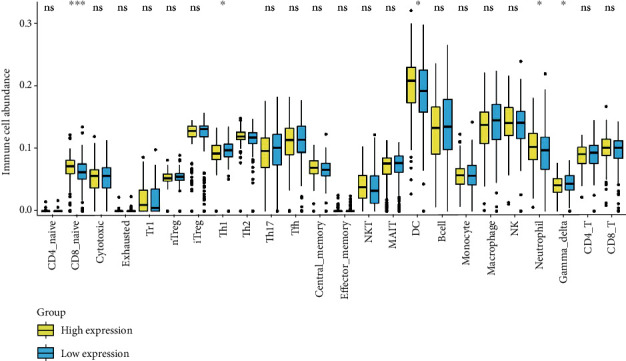
Immune cell abundance analysis between the low-GJA1-expression group and the high-GJA1-expression group. ^∗^*p* < 0.05, ^∗∗^*p* < 0.01, and ^∗∗∗^*p* < 0.001.

ImmuCellAI was used to analyze the infiltration difference of immune cells between the low- and the high-GJA1-expression group. We found that the abundance of CD8 naive, DC, and neutrophil is significantly increased in the high-GJA1-expression group, while the abundance of gamma delta and Th1 is significantly decreased in the low-GJA1-expression group **(**Figure 4**)**.

## 4. Discussion

The role of GJA1 in cancers has been widely studied in recent years. GJA1 has two opposite effects on cancers as it can act as an oncogene or a tumor suppressor gene. Many studies have unveiled the suppressive roles of GJA1 in tumorigenesis. In the colorectal cancer cell line (HT 29), Sirnes et al. found overexpression of GJA1 could inhibit Wnt signaling by interacting with *β*-catenin, thus inhibiting the growth of tumor cells [32]. Huang et al. identified that GJA1 could restrain glioblastoma development by reducing the anti-apoptotic Bcl-2 [13]. Similarly, overexpression of GJA1 was also found to inhibit the growth of lung cancer cells (LH7) [33]. Many studies have also reported the role of GJA1 in promoting cancer progression. Tang et al. reported that the overexpression of GJA1 could promote lymph node metastasis and peritoneal metastasis in gastric cancer [34, 35]. Zhao et al. found that high-GJA1 expression could indicate poor prognosis of gastric cancer patients based on five GEO datasets [18]. Ogawa et al. found that the silencing of GJA1 was associated with reduced invasion, migration, and metastasis [36]. From the Affymetrix analysis, Teleki et al. reported that high expression of GJA1 was associated with a reduced relapse-free survival (RFS) and overall survival (OS) in ER-negative breast cancer patients [37]. Moreover, the study by Stoletov et al. confirmed this observation using analysis from the Oncomine database [38]. Also, there are some researches on the impact of GJA1 on CC. Sun et al. found that HPV E6 may regulate GJA1 trafficking in cervical tumor cells, resulting in inhibition of the formation of gap junctions [39]. Using whole-genome microarray data, Cheng et al. showed that GJA1 was a critical gene for CC invasion and metastasis [40].

Using high-throughput RNA-sequencing data from the TCGA database, we aimed to explore the relationship between clinicopathological parameters and GJA1 expression and determine the role of GJA1 expression in CC progression, especially as a prognostic factor for CC. We also tried to screen signaling pathways related to GJA1 in CC to understand the underlying mechanism involved in the regulation of CC development by GJA1. In addition, we tried to explore GJA1 expression's impact on the abundance of the immune cells. First, we compared the expression of GJA1 in CC and normal tissues by GEPIA. The expression level of GJA1 in CC tissues was significantly lower than that in normal tissues. Six GEO datasets were used to verify this result, and the results from five datasets are the same as those from GEPIA. The HPA database also showed that the expression of GJA1 is higher in normal tissues than that in CC tissues at the protein level. These results suggest that GJA1 may be a tumor suppressor gene and play an important role in the progression of CC. In addition, GJA1 expression levels were different in groups classified by histology and FIGO stage. Further, chi-squared (*χ*^2^) test showed that the high expression level of GJA1 was significantly correlated with keratinization, hormone use, tumor size, and FIGO stage. Some literatures reported that GJA1 was associated with metastasis in a variety of tumors. Lin et al. found that the expression of GJA1 was higher in metastatic breast tumors than in primary breast tumors [41]. Wang et al. also showed that GJA1 could be used as a marker of metastasis in prostate cancer [42].

The Kaplan–Meier survival analysis showed that the high-GJA1-expression group indicated a worse prognosis than the low-GJA1-expression group. In the stratified analysis of SCC patients, early-stage patients (FIGO ≤ IIA2), and late-stage patients (FIGO ≥ IIB), we found the same result. Using five GEO datasets, Zhao et al. reported that high expression level of GJA1 was associated with poor prognosis in gastric cancer, which is similar to our result [18].

The univariate analysis showed that BMI, tumor size, lymph node, FIGO stage, lymphovascular invasion, distant metastasis, and high-GJA1expression were important predictors of survival. Importantly, multivariate analysis showed that tumor size, lymphovascular invasion, and the high-GJA1 expression were the important independent predictors of poor overall survival of CC. This result demonstrated the potential of GJA1 to become a prognosis biomarker of CC.

To further investigate the functions of GJA1 in CC, we performed GSEA using the TCGA data and 30 pathways were selected out based on established standards. The result showed that many cancer-related pathways, such as the p53 signaling pathway, the Wnt signaling pathways, and the MAPK signaling pathway, were enriched in the high-GJA1-expression group. Evidence has shown that the proliferation, migration, and invasion of CC cells can be modulated by regulating the p53 signaling pathway [43, 44]. Wnt signaling, which is needed for cell proliferation and differentiation, has been found to play a critical role in CC carcinogenesis [45, 46]. Activation of this pathway was shown to be related to the poor prognosis of CC patients [46]. MAPK signaling pathways regulate a variety of cellular activities, including proliferation, differentiation, survival, and death [47]. Many studies have reported that the interaction between HPV and the MAPK signaling pathway indirectly explains its role in CC [48, 49]. In addition, the TGF-*β* signaling pathway was enriched in the high-GJA1-expression group, which participates in the regulation of a variety of immune cells, such as NK cell, neutrophils, and macrophage [50]. Thus, we analyzed the differences of immune cells between the low-GJA1-expression group and the high-GJA1-expression group and found that the abundance of CD8 naive cell, DC, and neutrophil were significantly increased in the high-GJA1-expression group.

## 5. Conclusions

In conclusion, high GJA1 expression may be a potential prognostic molecular marker of poor survival in CC. Moreover, the p53 signaling pathway, Wnt signaling pathway, and MAPK signaling pathways may be the critical pathways regulated by GJA1. Moreover, GJA1 can affect the abundance of immune cells to a certain extent. Further experimental validation should be performed to prove the biologic impact of GJA1 in CC.

## Figures and Tables

**Table 1:** Clinical characteristics of patients with CC in TCGA database.

Clinical characteristics	Subgroup	Frequency	Percentage (%)
Total		304	
Age	Range:20-88 (average: 48.2, median: 46)		
BMI	Range: 13.4-70.5 (average: 27.9, median: 26.6)		
Histology	Squamous cell carcinoma (SCC)	252	89.0
	Adenomas and adenocarcinoma (ACC)	31	11.0
Keratinization	No	119	68.4
	Yes	55	31.6
Hormone use	No	89	56.7
	Yes	68	43.3
Hysterectomy	Other	8	4.8
	Hysterectomy	159	95.2
Tumor size	T1+T2	211	87.6
	T3+T4	30	12.4
Lymph node	N0	133	68.9
	N1	60	31.1
∗FIGO stage	≤IIA2 (early stage)	188	63.3
	≥IIB (late stage)	109	36.7
Differentiation grade	≤G2	153	56.3
	≥G3	119	43.7
Lymphovascular invasion	Absent	71	47.3
	Present	79	52.7
Distant metastasis	No	273	89.8
	Yes	31	10.2
Vital status	Alive	233	76.6
	Dead	71	23.4

∗FIGO stage: there were five samples only described as FIGO II without specific stages. We classified these samples as ≤IIA2 in this study. BMI: body mass index; FIGO: the International Federation of Gynecology and Obstetrics.

**Table 2:** Relationships between GJA1 expression and clinicopathological parameters in CC.

Clinicopathological parameters	GJA1 expression		Total	*p* value
	Low (*n* = 152)	High (*n* = 152)		
Age^∗^				
Median	46.0	46.5	46.0	0.485
Interquartile range	(38.0-55.0)	(38.0-59.0)	(38.0-57.0)	
BMI^∗^				
Median	26.9	25.8	26.6	0.246
Interquartile range	(23.1-33.3)	(22.6-31.2)	(22.6-32.4)	
Keratinization				
No	56	63	119	0.045
Yes	17	38	55	
Hormone use				
No	45	44	89	0.019
Yes	47	21	68	
Hysterectomy^#^				
Other	4	4	8	0.734
Hysterectomy	89	70	159	
Tumor size				
≤T2	119	92	211	0.042
≥T3	11	19	30	
Lymph node				
N0	79	54	133	0.063
N1	27	33	60	
FIGO stage				
≤IIA2	106	82	188	0.003
≥IIB	42	67	109	
Differentiation grade				
≤G2	74	79	153	0.151
≥G3	68	51	119	
Lymphovascular invasion				
No	40	31	71	0.697
Yes	42	37	79	
Distant metastasis				
No	141	132	273	0.088
Yes	11	20	31	

^∗^Mann-Whitney *U* test. ^#^Fisher's exact test.

**Table 3:** GJA1 expression correlated with clinicopathological parameters (logistic regression).

Clinicopathological parameters	Total (*N*)	OR	95% confidence interval		*p* value
			Lower limit	Upper limit	
Age (continuous)					
	304	1.007	0.991	1.024	0.393
BMI (continuous)					
	259	0.982	0.950	1.015	0.280
Keratinization					
Yes vs. no	174	1.987	1.011	3.905	0.046
Hormone use					
Yes vs. no	157	0.457	0.236	0.885	0.020
Hysterectomy					
Yes vs. no	167	0.787	0.190	3.257	0.740
Tumor size					
≥T3 vs. ≤T2	241	2.234	1.013	4.927	0.046
Lymph node					
N1 vs. N0	193	1.788	0.967	3.308	0.064
FIGO stage					
≥IIB vs. ≤IIA2	297	2.062	1.274	3.338	0.003
Differentiation grade					
≥G3 vs. ≤G2	272	0.703	0.434	1.138	0.151
Lymphovascular invasion					
Yes vs. no	150	1.137	0.597	2.165	0.697
Distant metastasis					
Yes vs. no	304	1.942	0.896	4.207	0.092

**Table 4:** Associations between overall survival and clinicopathological characteristics in CC using univariate analysis and multivariate analysis.

Parameters	Univariate analysis				Multivariate analysis			
	HR	95% CI		*p*	HR	95% CI		*p*
		Lower	Upper			Lower	Upper	
Age (continuous)	1.017	0.999	1.035	0.057	0.959	0.910	1.011	0.123
BMI (continuous)	0.953	0.911	0.996	0.034	0.976	0.894	1.066	0.593
Tumor size (≥T3 vs. ≤T2)	3.643	1.922	6.904	<0.001	6.181	1.219	31.334	0.028
Lymph node (N1 vs. N0)	2.695	1.358	5.349	0.005	2.256	0.636	8.008	0.208
FIGO stage (≥IIB vs. ≤IIA2)	1.861	1.166	2.970	0.009	0.317	0.042	2.403	0.266
Lymphovascular invasion (yes vs. no)	10.041	2.361	42.700	0.002	5.910	1.124	31.059	0.036
Distant metastasis (yes vs. no)	3.141	1.866	5.287	<0.001	1.043	0.257	4.240	0.953
*GJA1* expression level (high vs. low)	1.943	1.202	3.140	0.007	4.084	1.354	12.320	0.013

Likelihood ratio test = 28.37 on 8 df, *p* = 0.0004, *N* = 120, number of events = 18.

## Data Availability

Publicly available datasets were analyzed in this study. These can be found in The Cancer Genome Atlas (https://portal.gdc.cancer.gov/); the NCBI Gene Expression Omnibus (GSE39001, GSE52903, GSE63514, GSE6791, GSE7803, GSE9750).

## Supplementary Materials

Supplementary 1 Supplementary Figure 1: comparison of GJA1 expression between cervical cancer and normal using GEPIA and The Human Protein Atlas.

Supplementary 2 Supplementary Table 1: information of selected six GEO datasets and GJA1 expression in these datasets. Supplementary Table 2: gene sets enriched in the high-GJA1-expression phenotype.
