# Supplementary figures and images for: GJA1 Expression and Its Prognostic Value in Cervical Cancer
Source: Biomed Res Int. 2020 Nov 24;2020:8827920. doi: 10.1155/2020/8827920 (PMC7709497; doi:10.1155/2020/8827920)

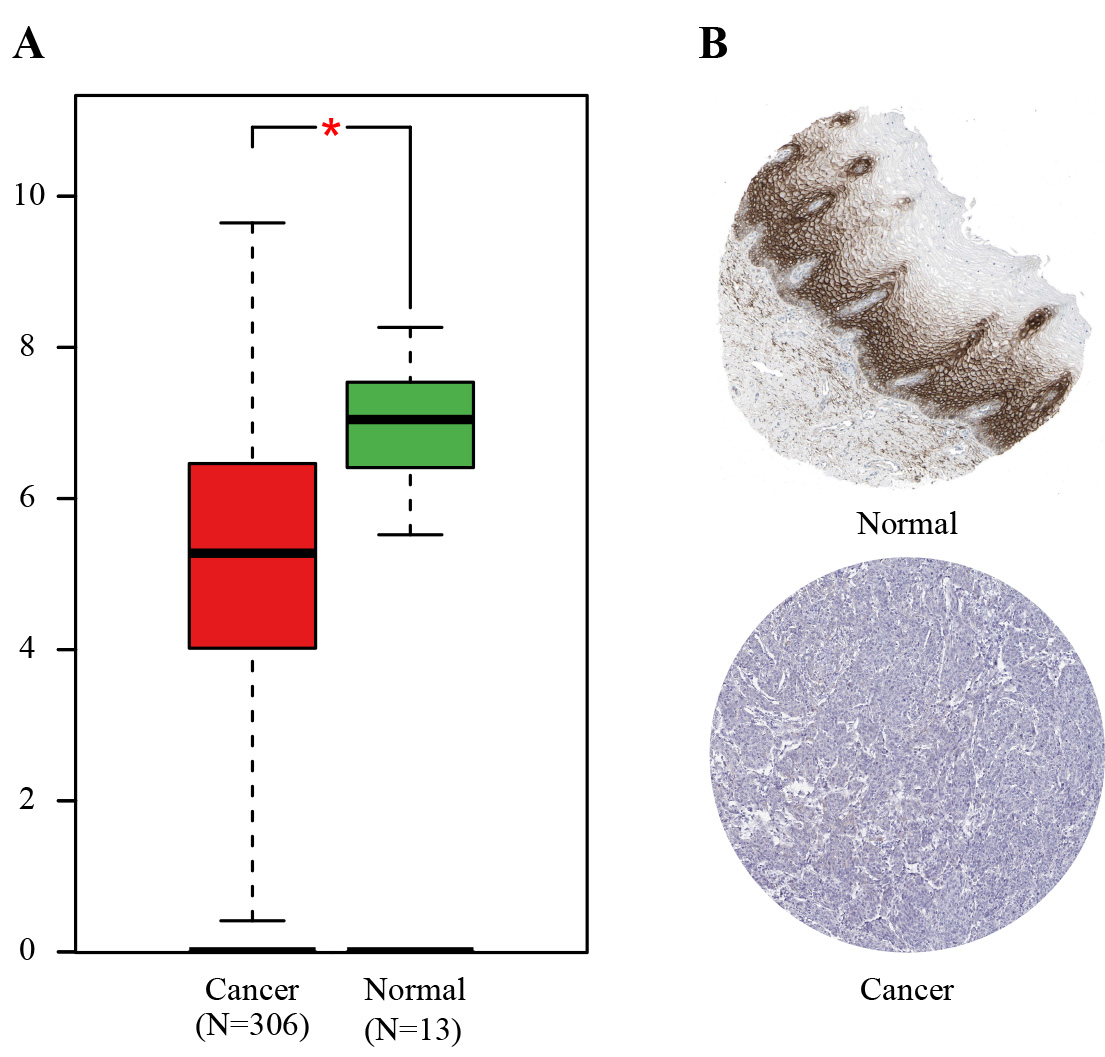

Supplement: Supplementary 1 — Supplementary Figure 1: comparison of GJA1 expression between cervical cancer and normal using GEPIA and The Human Protein Atlas. [file 8827920.f1.jpg]
