# Supplementary material for: GJA1 Expression and Its Prognostic Value in Cervical Cancer
Source: Biomed Res Int. 2020 Nov 24;2020:8827920. doi: 10.1155/2020/8827920 (PMC7709497; doi:10.1155/2020/8827920)
Supplement: Supplementary 2 — Supplementary Table 1: information of selected six GEO datasets and GJA1 expression in these datasets. Supplementary Table 2: gene sets enriched in the high-GJA1-expression phenotype. [file 8827920.f2.zip › Supplementary Table 1.docx]

Supplementary Table 1. Information of selected 6 GEO datasets and GJA1 expression in these datasets.

| **Datasets** | **Infomation of selected datasets** | | |  | **GJA1 expression*** | | | | | | |
| --- | --- | --- | --- | --- | --- | --- | --- | --- | --- | --- | --- |
|  | **Year** | **Platform** | **Cancer vs. Normal** |  | **logFC** | **AveExpr** | **t** | **P.Value** | **adj.P.Val** | **B** | **change** |
| GSE39001 | 2013 | GPL6244 | 19 vs. 5 |  | -1.808 | 9.216 | -3.452 | 1.98E-03 | 2.07E-02 | -1.630 | DOWN |
| GSE52903 | 2015 | GPL6244 | 55 vs. 17 |  | -1.628 | 8.862 | -6.022 | 6.21E-08 | 1.14E-06 | 7.817 | DOWN |
| GSE63514 | 2015 | GPL570 | 28 vs. 24 |  | -0.946 | 14.179 | -2.895 | 4.48E-03 | 1.60E-02 | -2.605 | NOT |
| GSE6791 | 2007 | GPL570 | 20 vs. 8 |  | -1.579 | 11.704 | -2.600 | 1.46E-02 | 2.49E-02 | -3.750 | DOWN |
| GSE7803 | 2007 | GPL96 | 21 vs. 10 |  | -1.898 | 12.873 | -4.022 | 2.54E-04 | 2.53E-03 | 0.097 | DOWN |
| GSE9750 | 2008 | GPL96 | 33 vs. 24 |  | -2.204 | 9.841 | -7.327 | 8.01E-10 | 3.07E-08 | 12.170 | DOWN |
| *Limma package was used to explore the relative expression of GJA1 between Cancer and Normal.  LogFC < -1 and adj.p.Val <0.05 was considered as significantly down. | | | | | | | | | |  |  |
