# Supplementary material for: GJA1 Expression and Its Prognostic Value in Cervical Cancer
Source: Biomed Res Int. 2020 Nov 24;2020:8827920. doi: 10.1155/2020/8827920 (PMC7709497; doi:10.1155/2020/8827920)
Supplement: Supplementary 2 — Supplementary Table 1: information of selected six GEO datasets and GJA1 expression in these datasets. Supplementary Table 2: gene sets enriched in the high-GJA1-expression phenotype. [file 8827920.f2.zip › Supplementary Table 2.docx]

Supplementary Table 2. Gene sets enriched in the high-GJA1 expression phenotype.

| **Gene set name** | **NES** | **NOM**  **p-val** | **FDR**  **q-val** |
| --- | --- | --- | --- |
| KEGG_P53_SIGNALING_PATHWAY | 2.33 | < 0.001 | 0.002 |
| KEGG_RENAL_CELL_CARCINOMA | 2.05 | < 0.001 | 0.035 |
| KEGG_NEUROTROPHIN_SIGNALING_PATHWAY | 2.03 | < 0.001 | 0.028 |
| KEGG_MELANOMA | 1.84 | 0.004 | 0.149 |
| KEGG_WNT_SIGNALING_PATHWAY | 1.83 | 0.004 | 0.122 |
| KEGG_PATHWAYS_IN_CANCER | 1.83 | 0.006 | 0.104 |
| KEGG_PANCREATIC_CANCER | 1.83 | 0.020 | 0.089 |
| KEGG_TGF_BETA_SIGNALING_PATHWAY | 1.79 | 0.004 | 0.116 |
| KEGG_SMALL_CELL_LUNG_CANCER | 1.77 | 0.018 | 0.121 |
| KEGG_COLORECTAL_CANCER | 1.74 | 0.024 | 0.135 |
| KEGG_BLADDER_CANCER | 1.74 | 0.014 | 0.126 |
| KEGG_ECM_RECEPTOR_INTERACTION | 1.74 | 0.002 | 0.117 |
| KEGG_AXON_GUIDANCE | 1.72 | 0.014 | 0.124 |
| KEGG_BASAL_CELL_CARCINOMA | 1.71 | 0.006 | 0.119 |
| KEGG_ERBB_SIGNALING_PATHWAY | 1.71 | 0.010 | 0.113 |
| KEGG_GNRH_SIGNALING_PATHWAY | 1.69 | 0.006 | 0.127 |
| KEGG_MAPK_SIGNALING_PATHWAY | 1.68 | 0.018 | 0.126 |
| KEGG_ADHERENS_JUNCTION | 1.67 | 0.038 | 0.123 |
| KEGG_INSULIN_SIGNALING_PATHWAY | 1.67 | 0.018 | 0.121 |
| KEGG_GLIOMA | 1.66 | 0.018 | 0.117 |
| KEGG_REGULATION_OF_ACTIN_CYTOSKELETON | 1.66 | 0.014 | 0.118 |
| KEGG_OLFACTORY_TRANSDUCTION | 1.65 | 0.002 | 0.117 |
| KEGG_GAP_JUNCTION | 1.64 | 0.013 | 0.124 |
| KEGG_ENDOMETRIAL_CANCER | 1.61 | 0.052 | 0.144 |
| KEGG_FOCAL_ADHESION | 1.6 | 0.047 | 0.142 |
| KEGG_MELANOGENESIS | 1.59 | 0.006 | 0.144 |
| KEGG_OOCYTE_MEIOSIS | 1.58 | 0.040 | 0.155 |
| KEGG_ADIPOCYTOKINE_SIGNALING_PATHWAY | 1.56 | 0.040 | 0.169 |
| KEGG_HEDGEHOG_SIGNALING_PATHWAY | 1.56 | 0.026 | 0.164 |
| KEGG_AMYOTROPHIC_LATERAL_SCLEROSIS_ALS | 1.54 | 0.042 | 0.178 |

Inclusion criteria: NES >1, NOM p-val < 0.05, FDR q-val < 0.25.
